# Chronic Glucocorticoid-Rich Milieu and Liver Dysfunction

**DOI:** 10.1155/2016/7838290

**Published:** 2016-08-11

**Authors:** Hernán Gonzalo Villagarcía, Vanesa Sabugo, María Cecilia Castro, Guillermo Schinella, Daniel Castrogiovanni, Eduardo Spinedi, María Laura Massa, Flavio Francini

**Affiliations:** ^1^Centro de Endocrinología Experimental y Aplicada (CENEXA), UNLP-CONICET-FCM, 1900 La Plata, Argentina; ^2^Cátedra de Farmacología Básica, Facultad de Ciencias Médicas, UNLP and CICPBA, 1900 La Plata, Argentina; ^3^Instituto Multidisciplinario de Biología Celular (IMBICE), CONICET-CICPBA-UNLP, 1900 La Plata, Argentina

## Abstract

We investigated the impact of chronic hypercorticosteronemia (due to neonatal monosodium L-glutamate, MSG, and treatment) on liver oxidative stress (OS), inflammation, and carbohydrate/lipid metabolism in adult male rats. We evaluated the peripheral concentrations of several metabolic and OS markers and insulin resistance indexes. In liver we assessed (a) OS (GSH and protein carbonyl groups) and inflammatory (*IL-1b*,* TNFa*, and* PAI-1*) biomarkers and (b) carbohydrate and lipid metabolisms. MSG rats displayed degenerated optic nerves, hypophagia, low body and liver weights, and enlarged adipose tissue mass; higher peripheral levels of glucose, triglycerides, insulin, uric acid, leptin, corticosterone, transaminases and TBARS, and peripheral and liver insulin resistance; elevated liver OS, inflammation markers, and glucokinase (mRNA/activity) and fructokinase (mRNA). Additionally, MSG liver phosphofructokinase-2, glucose-6-phosphatase (mRNA and activity) and glucose-6-phosphate dehydrogenase,* Chrebp*,* Srebp1c*, fatty acid synthase, and glycerol-3-phosphate (mRNAs) were increased. In conclusion adult MSG rats developed an insulin-resistant state and increased OS and serious hepatic dysfunction characterized by inflammation and metabolic signs suggesting increased lipogenesis. These features, shared by both metabolic and Cushing's syndrome human phenotypes, support that a chronic glucocorticoid-rich endogenous environment mainly impacts on hepatic glucose cycle, displacing local metabolism to lipogenesis. Whether correcting the glucocorticoid-rich environment ameliorates such dysfunctions requires further investigation.

## 1. Introduction

Several studies have described the effects of neonatal monosodium L-glutamate (MSG) administration in rodents [1, 2]. It is openly accepted that MSG treatment induces morphological, behavioral, and endocrine abnormalities, such as growth disturbances, hyperadiposity, and hypogonadism [1, 3]. Other studies reported severe loss of catecholaminergic and peptidergic neurons in the retina and the hypothalamic arcuate nucleus (ARC) [3–5]. This neuronal loss alters several endocrine-metabolic functions, such as energy balance [6–8] and anterior pituitary hormone secretion [9, 10]. A conspicuous effect of MSG-induced hypothalamic damage is enhanced overall response of median eminence neurons terminals [9, 10]. The extensive brain damage has been used to explain several of the altered neuroendocrine functions in this model [5, 11]. ARC plays a pivotal role in regulation of energy storage and expenditure, and adipose tissue leptin secretion and hypothalamic leptin signaling system play key roles for maintaining homeostasis [12].

Among others, neonatal MSG treatment also affects white adipocyte function. MSG rats develop hyperleptinemia due to both enlarged adipose tissue mass and adipocyte size [13]. Moreover, chronic excess of endogenous glucocorticoids (GC) has been considered a main factor of the hypertrophic adiposity developed by the adult MSG rat [14]. Indeed, MSG animals are partly refractory to leptin inhibition (leptin resistance) of food intake and body weight gain [15] and, importantly, to the leptin negative control on adrenocortical function [16–18]. Moreover, the adult MSG rat phenotype is also characterized by an enhanced proinflammatory peripheral environment [19].

Because of the overall endocrine-metabolic characteristics, namely, hypercorticosteronemia and adipose tissue mass excess, the MSG rat is an excellent experimental model for basic investigation of different dysfunctions representative of the human Cushing's syndrome (hypothalamic obesity) phenotype [13, 14, 16–19]. Interestingly, it has been addressed that correcting their high circulating levels of GC various neuroendocrine and adiposity dysfunctions can be reversed [16, 18], including the hypertrophy of abdominal white adipocytes [14].

Endocrine and metabolic dysfunctions characterizing the hypothalamic obese rat phenotype have been largely revisited by several authors including us; however, whether a liver shift from carbohydrate to lipid metabolism sustaining liver/ectopic lipid deposition exists in MSG rats has not been addressed yet. Therefore, in the present study we examined whether neonatal MSG treatment later results in hepatic metabolism dysfunction by analysing local carbohydrate and lipid metabolic pathways. In addition, the peripheral concentrations of several metabolic-endocrine markers and tissue insulin sensitivity, oxidative stress (OS), and inflammation were evaluated.

## 2. Materials and Methods

### 2.1. Chemicals and Drugs

Reagents of the purest available grade were obtained from Sigma Chemical Co. (St. Louis, MO, USA). Primary antibodies were obtained from Santa Cruz Biotechnology, Inc. (Santa Cruz, California, USA). The secondary antibody, peroxidase-conjugated AffiniPure donkey anti-rabbit IgG, was provided by Dianova (Hamburg, Germany).

### 2.2. Experimental Animals

Adult male and female Wistar rats were allowed to mate in colony cages in a light (lights on from 07:00 to 19:00 h) and temperature (20–22°C) controlled room. Rat chow and water were available* ad libitum*. Pregnant rats were transferred to individual cages. Beginning on day 2 after parturition, newborn male pups were injected i.p. with either 4 mg/g BW MSG (Sigma Chemical Co., St. Louis, MO) dissolved in sterile 0.9% (w/v) NaCl or 10% (w/v) NaCl (littermate controls; C) once every two days up to 10 days of age [14]. Rats were weaned at 21 days of age and housed (3 rats per cage) in a controlled environment (20–22°C and lights on from 07:00 to 19:00 hs) with free availability to rat Purina chow and water. Thereafter, individual daily body weight and food intake were recorded until the experiment day (150 days of age). In each experiment, control (C) and MSG rats were members of the same litters; however, when accumulating experiments, each different experiment was performed with animals from different litters. On the morning (between 08:00 and 10:00 h) of the experiment day, overnight-fasting animals were weighed and euthanized and trunk blood was collected (into EDTA-coated tubes). The brain was immediately dissected out in order to check effectiveness of MSG treatment by macroscopic observation of degenerated optic nerves (inclusion criteria). Thereafter, visceral adipose tissue (VAT) pad and the liver were dissected and weighed. Next, hepatic medial lobes were excised to perform several other biochemical assays (see below). Animals were killed by decapitation according to protocols for animal use, in agreement with NIH Guidelines for care and use of experimental animals. All experimentation was approved by our Institutional Animal Care Committee.

### 2.3. Circulating Metabolites and Insulin Sensitivity Indexes

Glycemia was measured by the glucose-oxidase GOD-PAP method (Roche Diagnostics, Mannheim, Germany) and plasma concentrations of triglycerides, uric acid, and transaminases aspartate aminotransferase (GOT) and alanine aminotransferase (GPT) were determined by commercial (enzymatic-colorimetric) assays (Wiener Lab, Argentina). Peripheral immunoreactive insulin [13], leptin, and corticosterone [16] levels were determined by previously described specific radioimmunoassays. Lipid peroxidation was estimated by measuring TBARS (thiobarbituric acid-reactive substance) [20]. The amount of TBARS formed was calculated by extinction coefficient of the MDA (malondialdehyde)-TBA complex of 1.56 × 105 (mol/L)^−1^·cm^−1^ and expressed as pmol of MDA/mg of plasma protein per mL of plasma, measured with the Bio-Rad Protein Assay kit.

Fasting glycemia and insulin values were used to estimate peripheral insulin resistance by homeostasis model assessment-insulin resistance (HOMA-IR) [insulin (*μ*UI/mL) × glycemia (mM)/22.5]. Liver insulin sensitivity index (LISI) was calculated with the following formula: *k*/(fasting plasma insulin) × fasting glycemia, where *k* = 22.5 × 18 [21].

### 2.4. Liver Protein Carbonyl Groups and Reduced Glutathione (GSH)

Protein carbonyl and GSH liver contents were determined as described elsewhere [22]. Briefly, both components were spectrophotometrically measured (at 366 nm for protein carbonyl groups and 414 nm for GSH) and results were expressed in nmol of carbonyl residues per mg of protein based on the molar extinction coefficient of 21,000 M^−1 ^cm^−1^. The results of GSH content were expressed in *μ*mol of –SH per g of tissue.

### 2.5. Total RNA Isolation

Total liver RNA was isolated using the TRIzol Reagent (Gibco-BRL, Rockville, MD, USA) as previously described [22]. The integrity and quality of the isolated RNA were checked by agarose-formaldehyde gel electrophoresis and by measuring the 260/280 nm absorbance ratio. DNA contamination was avoided by using DNase I digestion reagent (Gibco-BRL). Reverse transcription-PCR was performed with SuperScript III (Gibco-BRL) and total RNA (50 ng) as a template.

### 2.6. Liver mRNA Expression Levels Using Real-Time PCR (qPCR)

qPCR was performed with a Mini Opticon Real-Time PCR Detector Separate MJR (Bio-Rad), using SYBR Green I as fluorescent dye. cDNA (10 ng) was amplified in a qPCR reaction mixture containing 0.36 *μ*M of each specific primer, 3 mM MgCl_2_, 0.2 mM dNTPs, and 0.15 *μ*L Platinum Taq DNA Polymerase (6 U/*μ*L) (Invitrogen). Samples were first denatured at 95°C for 3 min followed by 40 PCR cycles. Each cycle comprised a melting step at 95°C for 30 sec, an annealing step at 65°C for 30 sec, and an extension step at 72°C for 45 sec followed by a final extension at 72°C for 10 min. Specific oligonucleotide primers (Invitrogen) are shown in Table 1. Amplicons were designed in a size range of 90 to 250 bp, with* Actinb* used as housekeeping gene. Purity and specificity of amplified PCR products were verified by performing melting curves generated at the end of each PCR. Data are expressed as relative gene expression after normalization to* Actinb* using Qgene96 and LineRegPCR software.

### 2.7. Western Blot Analysis

Immunodetection of glucokinase (GCK), fructokinase (FK), phosphofructokinase-2 (PFK2), and ACTINB was performed with liver homogenates from each experimental group. Protein concentration was quantified by the Bio-Rad Protein Assay kit. Thereafter, dithiothreitol and bromophenol blue were added (final concentrations 100 mM and 0.1% w/v, resp.). Aliquots of 20 *μ*g for GCK and 100 *μ*g for PFK2 of whole protein were placed in reducing 10% (w/v) SDS-PAGE and electroblotted to polyvinylidene difluoride membranes. ACTINB density was used to normalize protein content: the relative content of target protein was divided by the relative ACTINB protein level in each group. Nonspecific binding sites of membranes were blocked by overnight incubation with nonfat dry milk at 4°C. Enzyme identification and quantification were performed with specific primary antibodies against FK and GCK (final dilution of 1 : 2000) [22] for 90 min, PFK2 antibody (final dilution of 1 : 10000) for 16 hours [23], and ACTINB antibody (final dilution of 1 : 10000) for 60 min. After the respective incubation period, membranes were rinsed in TBS and further incubated (1 h) with the corresponding secondary antibody: for GCK and PFK2, anti-sheep IgG streptavidin-peroxidase conjugate and anti-chicken IgY peroxidase-labelled were used, respectively, and for ACTINB biotinylated anti-mouse IgG was used. Diaminobenzidine (DAB, Sigma Co.) was used for color development. Bands were quantified by densitometry using Gel-Pro Analyser software.

### 2.8. Glucokinase Activity

Freshly removed liver pieces were immediately homogenized in hand-held homogenizers (20 times) containing ice cold phosphate saline buffer, with 0.1 mM PMSF, 0.1 mM benzamidine, 2 mM DTT, 4 *μ*g/mL aprotinin, and 0.3 M sucrose (pH 7.5). Homogenates were then passed through a 23-gauge needle syringe (5 times) to ensure appropriate sample mixing. Aliquots of homogenates were centrifuged at 600 ×g to separate and discard the nuclear fraction. Supernatants were centrifuged at 8,000 ×g and 100,000 ×g at 4°C, collected, and identified as cytosolic fractions. Phosphorylation in the 100,000 ×g soluble cytosolic fractions was measured at 37°C, pH 7.4, by recording increasing absorbance (measured at 340 nm) in a well-established enzyme-coupled photometric assay containing glucose-6-phosphate dehydrogenase (G6PDH), ATP, and NADP [23]. For each assay, five different experiments were performed in triplicate. GCK activity was calculated by subtracting activity measured at 1 mM glucose (hexokinase) from that measured at 100 mM glucose. Enzyme activity was then expressed in mU per milligram of protein. One unit of enzyme activity was defined as 1 *μ*mol glucose-6-phosphate formed from glucose and ATP per minute at 37°C.

### 2.9. Glucose-6-Phosphatase (G6Pase) Activity

Homogenization of liver samples and isolation of local microsomes were done as described by Nordlie and Arion [24]. Briefly, the homogenization medium was 0.25 M sucrose/5 mM Tris-acetate/0.5 mM Na-EDTA, pH 7.4 (3 mL per g of tissue). Microsomes were washed once with 0.25 M sucrose/5 mM Tris-acetate, pH 7.4, and centrifuged at 100,000 ×g. Untreated microsomes were diluted to the desired final concentration with the sucrose buffered solution and then assayed without any other further treatment. Fully disrupted microsomes were prepared at 0°C by adding 0.1 mL of 0.75% (w/v) Triton X-100 and 0.9 mL of untreated microsomes (containing approximately 10 mg of protein) and allowed to stand in an ice bath for 20 min. The reaction was stopped by adding 250 *μ*L of 10% (w/v) TCA and 2 mL of MoNH_4_ (diluted in H_2_SO_4_ 1 N) plus 320 *μ*L of FeSO_4_ (diluted in H_2_SO_4_ 0.15 N) to 200 *μ*L of sample. The OD at 660 nm was determined photometrically and results were expressed as “latency.” Latency was calculated by the following formula: 100 × (activity measured in disrupted microsomes minus activity measured in untreated microsomes)/activity measured in disrupted microsomes [25].

### 2.10. Fructokinase Activity

Pieces of liver were homogenized in buffer containing 25 mM HEPES (pH 7.1), 100 mM KCl, 1 mM DTT, and 0.1 mM EDTA and spun at 10,000 ×g at 4°C for 20 min. We measured FK activity by using a coupled enzymatic assay based on existing methods [26]. Briefly, 20 *μ*L of the sample was added to 200 *μ*L of the reaction mixture containing 25 mM HEPES (pH 7.1), 6 mM MgCl_2_, 25 mM KCl, 10 mM NaF, 5 mM D-fructose, 0.2 mM NADH, 1 mM phosphoenolpyruvate, 40 U/mL pyruvate kinase, 40 U/mL lactate dehydrogenase, and 50 mM N-acetyl-D-glucosamine (to inhibit hexokinase activity). The reaction was started by adding 10 *μ*L of ATP (5 mM final concentration) and quantitatively measured according to changes in optical density at 340 nm (30 min).

### 2.11. Statistical Analysis

Statistical analysis was performed by ANOVA, followed by Dunnett's test for multiple comparisons with the Prism analysis program (GraphPad). Bartlett's test was used to assess variance homogeneity. Results were expressed as means (±SEM) of the indicated number of observations; differences were considered significant when *P* was less than 0.05 [22].

## 3. Results

### 3.1. The MSG Rat Phenotype

On the experimental day, MSG rats showed degenerated optic nerves (macroscopic observation) and other characteristics of this model, such as (a) low body weight, (b) high visceral adipose tissue mass, and (c) hypophagia (average of individual daily food intake between 60 and 150 days of age), thus confirming several features of the complete MSG phenotype (Table 2). Moreover, relative wet liver weight (expressed as individual liver weight per 100 g body weight) was also significantly (*P* < 0.05 versus C rats) reduced in MSG rats (Table 2).

### 3.2. Circulating Metabolites and Insulin Sensitivity Indexes

MSG rats had significantly (*P* < 0.05 versus C rats) higher plasma insulin and triglyceride levels, despite similar glycemia (Table 2). Uric acid levels in MSG animals also were higher (*P* < 0.05 versus C values), as well as those of GOT and GPT. These altered parameters are clearly indicative of liver dysfunction (Table 2). Regarding two specific peripheral markers in this animal model, leptin and corticosterone concentrations, we found both hormones several times higher (*P* < 0.05 versus C values) in MSG rats (Table 2).

Finally, after calculation, HOMA-IR and LISI values were significantly (*P* < 0.05) higher and lower, respectively, in MSG (11.35 ± 0.98 and 1.99 ± 0.19, resp.) than in C (5.35 ± 0.46 and 3.24 ± 0.31, resp.) animals (*n* = 12 rats per group).

### 3.3. Peripheral and Liver Oxidative Stress Markers

Circulating levels of TBARS, a marker of peripheral OS, were significantly (*P* < 0.05 versus C values) higher in MSG animals (Table 3). Concordant with this finding, liver content of protein carbonyl groups also was significantly (*P* < 0.05 versus C) higher in MSG rats (Table 3), whereas the local amount of GSH, a peptide highly protective against OS development, was significantly (*P* < 0.05 versus C) lower in MSG rats (Table 3).

### 3.4. Neonatal MSG Treatment Resulted in Liver Inflammation and Dysmetabolism

**Figure 1:**
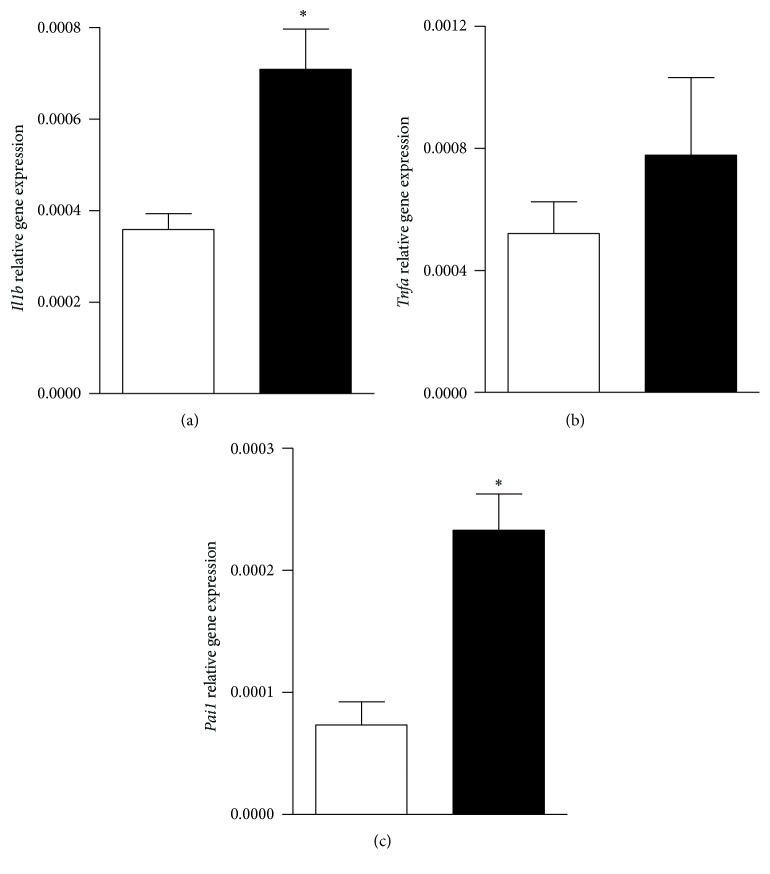
Liver mRNA levels of* Il1b*,* Tnfa*, and* Pai-1* (panels (a), (b), and (c), resp.) in control (white bars) and MSG rats (black bars). Results are means ± SEM (*n* = 8 rats per group). ^*∗*^ *P* < 0.05 versus control values. Statistical analysis was performed by ANOVA, followed by Dunnett's test for multiple comparisons.

The livers from MSG rats displayed a significant (*P* < 0.05 versus C values) increase in the mRNA levels of* Il1b*,* Tnfa*, and* Pai-1* (Figures 1(a), 1(b), and 1(c), resp.), all key signals indicative of local inflammatory process.

**Figure 2:**
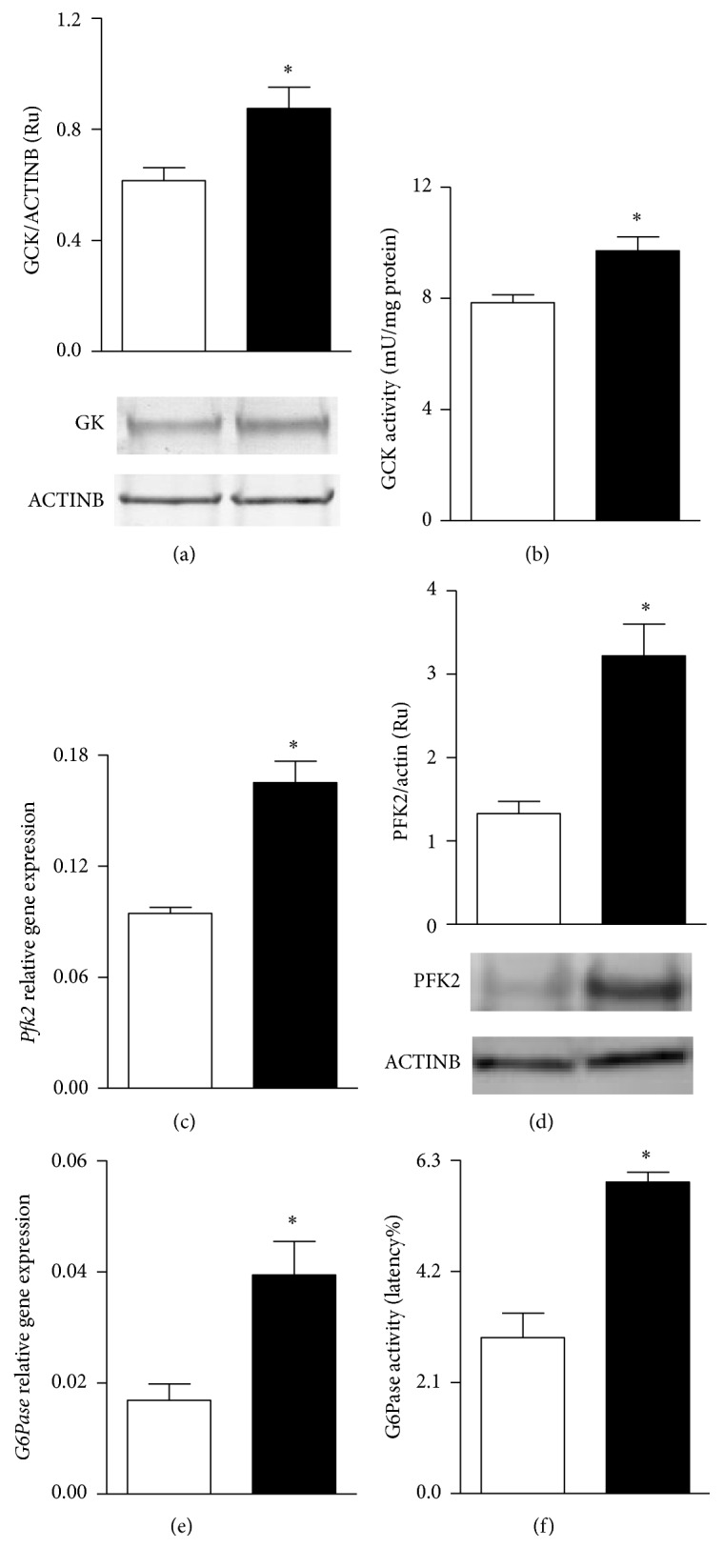
Hepatic protein content and activity levels of GCK (panels (a) and (b), resp.), mRNA levels and protein content of PFK2 (panels (c) and (d), resp.), and mRNA levels and enzyme activity of G6Pase (panels (e) and (f), resp.) in control (white bars) and MSG-treated rats (black bars). Results are means ± SEM (*n* = 8 rats per group). ^*∗*^ *P* < 0.05 versus control values. Statistical analysis was performed by ANOVA, followed by Dunnett's test for multiple comparisons.

Although no group difference was observed in hepatic glycogen content (data not shown), MSG livers showed a significantly higher* Gck* mRNA expression (1.77 ± 0.31 versus 1.01 ± 0.26 AU; *P* < 0.05 versus C; *n* = 8 rats per group). Moreover, GK protein content and activity were also significantly (*P* < 0.05 versus C) higher in MSG livers (Figures 2(a) and 2(b), resp.). Tissues from MSG rats displayed a robust increase (*P* < 0.05 versus C) in PFK2 mRNA expression and protein content (Figures 2(c) and 2(d), resp.); similarly,* G6Pase* mRNA expression and activity also were significantly (*P* < 0.05 versus C) higher in MSG tissues (Figures 2(e) and 2(f), resp.).

**Figure 3:**
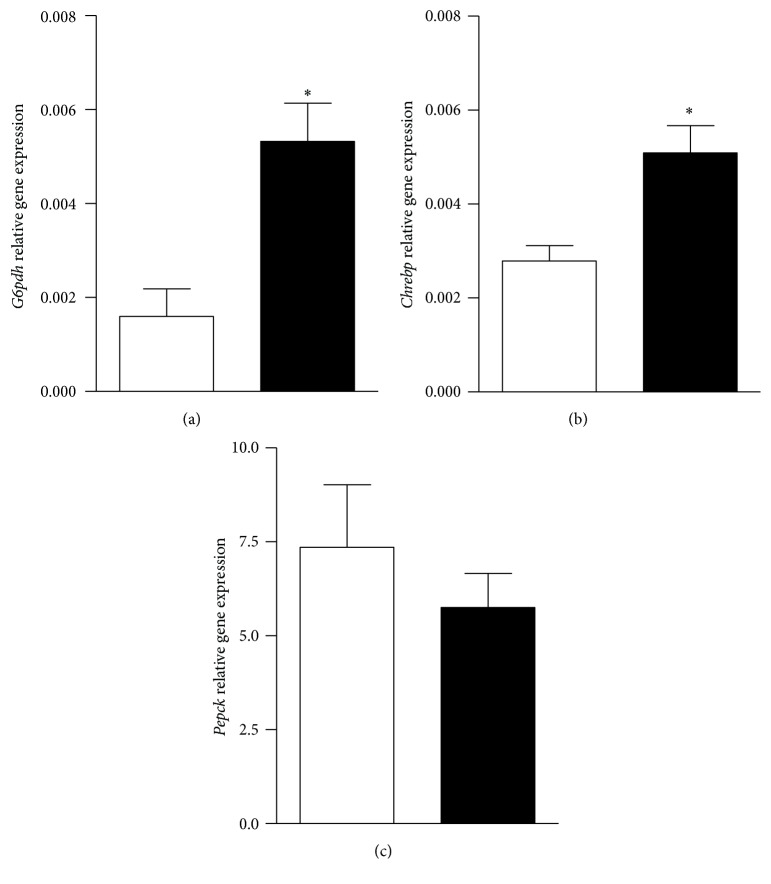
Hepatic* G6pdh* (panel (a)),* Chrebp* (panel (b)), and* Pepck* (panel (c)) gene expression in control (white bars) and MSG-treated rats (black bars). Results are means ± SEM (*n* = 8 rats per group). ^*∗*^ *P* < 0.05 versus control values. Statistical analysis was performed by ANOVA, followed by Dunnett's test for multiple comparisons.

The mRNA levels of* G6pdh* and* Chrebp* were also significantly (*P* < 0.05 versus C) increased in MSG tissues (Figures 3(a) and 3(b), resp.), despite no group difference in that of phosphoenolpyruvate carboxykinase (*Pepck*) (Figure 3(c)).

**Figure 4:**
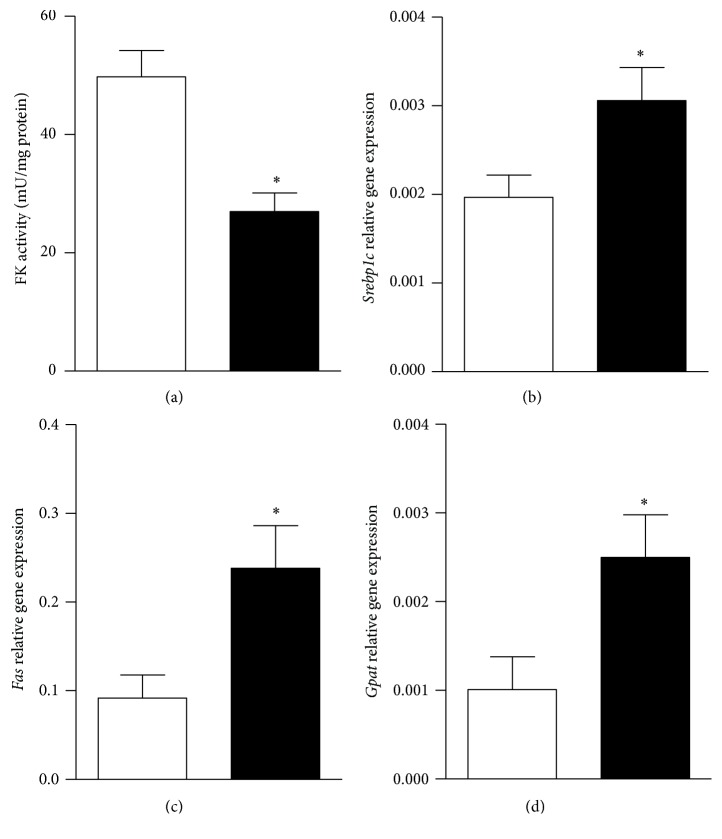
Liver FK activity (panel (a)), mRNA levels of* Srebp1c* (panel (b)),* Fas* (panel (c)), and* Gpat* (panel (d)) in control (white bars) and MSG rats (black bars). Results are means ± SEM (*n* = 8 rats per group). ^*∗*^ *P* < 0.05 versus control values. Statistical analysis was performed by ANOVA, followed by Dunnett's test for multiple comparisons.

Finally, regarding the lipogenic pathway, FK mRNA expression and protein content were significantly (*P* < 0.05 versus C values) augmented in MSG tissues (1.93 ± 0.16 versus 1.02 ± 0.12 AU and 4.69 ± 0.58 versus 2.11 ± 0.49 AU, resp.; *n* = 8 rats per group). Interestingly, FK activity was drastically (*P* < 0.05 versus C) reduced in livers from MSG animals (Figure 4(a)). Moreover, liver lipogenesis-related genes expressions were modified by the neonatal MSG treatment; indeed the liver mRNA expression levels of* Srebp1c*,* Fas*, and* Gpat* were 2-fold higher (*P* < 0.05 versus C) in MSG tissues (Figures 4(b), 4(c), and 4(d), resp.).

## 4. Discussion

The hypothalamus is a key brain structure in charge of the control of energy balance, and its injury is a major cause of neuroendocrine (hypothalamic) obesity development [27]. Conspicuous neuronal loss at the hypothalamic level can certainly be induced by the neonatal administration of MSG [28]. As mentioned above, in adult age, MSG-damaged rats have already installed hypophagy, low body weight, hyperinsulinemia, hyperleptinemia, hyperuricemia, hyperadiposity, inflammation, and increased plasma levels of corticosterone, TBARS, and lipids [19, 29]. In the present study we were able to find that neonatal MSG treatment induced a wide neuroendocrine-metabolic alteration once adult age was reached. It must be borne in mind that one of the expected consequences of neonatal MSG treatment in rats is the development of stunted growth, thus reaching adult age with a paradoxical phenotype characterized by hyperadiposity [13, 14, 16–19] and decreased body and several tissues weights [3]. Chronic excess of endogenous GC, in turn, will induce protein breakdown (chronic hypercatabolic state), thus resulting in hyperuricemic phenotype [30]. Both metabolic and Cushing's syndrome human phenotypes [30, 31] share hyperuricemia, and it has been claimed that this high uric acid peripheral level may have a key pathogenic role in several metabolic syndrome phenotypes [32], because, among others, uric acid peripheral levels correlate well with those of insulin and triglycerides in fructose-induced metabolic syndrome and, interestingly, correcting the IR state the hypertriglyceridemia and hyperuricemia can be overridden [32]. Aside from the disturbances mentioned above, we determined in this rat phenotype a deeply dysfunctional liver, as indicated by wet tissue weight, carbohydrate and lipid metabolisms, and local increase in OS and inflammatory markers. It should be stressed that in the adult male MSG rat no previous integrative hepatic studies on the carbohydrate and lipid metabolic functions and the inflammatory and OS processes have been examined.

As shown by the endogenous OS status, the higher HOMA-IR and lower LISI values, this being the first work demonstrating the latter, recorded in MSG animals clearly indicate that these rats developed OS-related peripheral and liver IR. However, it remains to be precisely ascertained whether long-term exposure to high circulating leptin levels [14] could be a key cooperative factor for the establishment of an overall IR state [13, 28], namely, at the liver level. It is also plausible that, as occurred at the adrenal level [13, 16], liver IR could be reversed by correcting endogenous overproduction of glucocorticoid and leptin.

High glucocorticoid treatment is known to induce cell inflammation and to enhance OS in hepatocytes by increasing and decreasing total oxidative and antioxidant capacities, respectively [33]. High OS in the MSG liver could result from endogenous enhanced reactive oxygen species production and/or deficient antioxidant mechanism [34, 35], consequently designating high OS as a key pathogenic factor in the initiation and progression of metabolic diseases, including hepatic steatosis [36] and type 2 diabetes [34, 37]. Strong evidence also indicates that intracellular signals generated by OS may also induce chronic inflammation together with insulin resistance* in vivo* [38, 39]. High fat diet intake in rodents is able to increase OS at both liver and adipose tissue levels [40], and Bloch-Damti et al. [41] claimed that local OS increased IRS-1 and IRS-2 serine phosphorylations, decreased IRS-1 protein content, and impaired insulin sensitivity in 3T3-L1 adipocytes. Interestingly, Park et al. [42] also observed that MSG administration in rodents reduces liver antioxidant biomarkers, such as glutathione level and activities of various antioxidant enzymes, also enhancing local TBARS content.

We found that our MSG rats are dyslipidemic, tallying with the stimulated liver lipogenic process revealed by elevated expression levels of their master regulator gene,* Srebp1c*, as well as their target genes,* Fas* and* Gpat* [43]. Although other authors have reported similar changes in lipogenic genes [44], we also found an enhanced gene expression and reduced activity in liver FK, enzyme providing fructose-1-P that, in turn, renders trioses for entering into triglyceride synthesis. This phenomenon clearly indicates that when needed substrates are available, local synthesis of triacylglycerols is expected [45, 46].

It should be considered that, within an insulin-resistant state scenario, we were able to determine that, in chronic hypercorticosteronemic MSG rats, similarly to that previously found in Cushing's patients [47], the liver high activity of the two key enzymes, GCK and G6Pase, assures (within a very active futile cycle) a drastic increase of high glucose liver content. Although a previous study has reported an enhanced activity of G6Pase in MSG animals [48], to our knowledge this is the first report of an active involvement of GCK, the hepatic glucose sensor, in the changes induced by MSG. These data are in agreement with and supported by those indicating that hypercortisolemic-hyperinsulinemic (IR) patients did not develop hyperglycemia due to their reduced peripheral glucose uptake [47]. Indeed, our MSG rats, sharing with those patients the high GC and insulin peripheral levels among other conditions, were fully able to maintain a normal glycemia. These data probably indicate that GC, through a liver glucocorticoid receptor- (GR-) mediated effect [49], could be responsible for further distribution of the intrahepatic glucose store. In this regard, if glucose did not enter in the MSG liver glycogenogenic process, indeed we and other researchers [50] did not notice any group difference in liver glycogen content. The fact that this cycle is highly active in our normoglycemic MSG rats suggests that glucose cycling could be a common feature of the metabolic syndrome and in nonhyperglycemic Cushing's syndrome phenotypes [47, 51]; moreover, the enhanced glucose cycle activity has been claimed to counteract hyperglycemia in hyperglycemic Cushing's syndrome patients [47], ob/ob mice [52], and diabetic rats [53]. Because glucose-6-phosphate (G6P) is a branching point for glucose fate, it is also plausible to expect that glucose is metabolized through either or both of the following pathways: (a) being channeled into the hexose monophosphate shunt pathway due to enhanced G6PDH activity, thereby producing cell reducing power (as NADPH) later used for hepatocyte lipogenesis and replenishing GSH pool, hence providing an additional mechanism to counteract OS [54], and/or (b) G6P being isomerized to fructose-6-phosphate (F6P), then oxidized to pyruvate and acetyl-CoA, and finally entering into the lipid anabolic pathway. This/these possibility/ies could be reinforced by our finding of a robust increase in PFK2, a potent cytosolic activator of GCK [23] in the MSG liver, thus suggesting that, in this model, there is an active role of the posttranslational regulatory mechanisms of GCK activity. It is known that GCK is also expressed in neurons, pituitary cells, pancreatic B-cells, and enteroendocrine K and L cells [55], an overall cell network acting as a glucose sensor for maintaining glucose homeostasis. Therefore, further research focused on the fact that whether or not the neonatal MSG treatment broadly impairs this glucose sensor net is required.

MSG treatment also yielded increased liver* Chrebp* gene expression, a parameter well correlating with increased expression of glycolysis-related genes and a key factor in the activation of promoter genes involved in the lipogenic process. In fact,* Chrebp* has been designated as a central link between these two pathways in hepatocytes [56]. Kabashima et al. [57] reported that enhanced activity of the hexose monophosphate shunt pathway leads to intrahepatocyte increase in xylulose-5-phosphate, which in turn activates phosphoprotein phosphatase A2, a main activator of* Chrebp*. Our results showing high gene expression of* G6pdh*, a rate limiting enzyme of the hexose monophosphate shunt pathway, demonstrate for the first time that the abovementioned mechanisms remained fully operative in MSG animals. Additionally, as mentioned above, we noticed that FK protein and activity were drastically augmented and reduced, respectively, in livers from MSG rats. It must be borne in mind that the liver lipid metabolism in MSG rats is clearly displaced to enhanced lipogenesis; thus it could be speculated that, in the postprandial condition any further liver entry of hexoses, through FK downstream reactions, could result in dramatic local lipid overproduction because carbons enter through this pathway bypass glycolysis regulatory steps and are then used in the lipogenic process in a direct and an uncontrolled fashion [45–47]. Therefore, it could be hypothesized that inhibited liver FK activity could represent a novel adaptive (protective feedback) mechanism in the MSG liver against further detrimental overlipogenic process in hepatocytes (e.g., hepatic steatosis). In spite of chronic endogenous glucocorticoid excess in MSG rats, it has been clearly established that white adipocytes isolated from these rats are partly refractory to dexamethasone-elicited leptin secretion and that adipocyte GC resistance can be fully reversed shortly after correction of endogenous GC overproduction [13]. Hence, it is also possible that endogenous GC-rich environment in MSG rats could be responsible for liver dysmetabolism [58]. In fact, we recently noticed that, after three weeks of bilateral adrenalectomy, adult male MSG rats normalized their plasmatic concentrations of leptin, triglyceride, uric acid, and transaminases (data not shown). However, because MSG animals developed hyperleptinemia early in life (30 days of age) [14], a detrimental leptin effect on liver metabolism must not be discarded. In this regard, it has previously been assessed that livers from DIO mice are highly susceptible to inflammation, OS, and nonalcoholic hepatic steatosis development; conversely, leptin KO-DIO mice are protected from these dysfunctions [59]. It must be considered that MSG rats also developed an early peripheral leptin resistance [13], a dysfunction fully reversed after normalization of endogenous GC environment [13]; therefore, this leptin-resistance condition could be contributing to liver carbohydrate dysmetabolism. Altogether, our data strongly suggest that adult, neonatally damaged, MSG rats are able to remain prediabetic (normal basal glycemia) due to enhanced entry of glucose into the liver (high glucose cycle activity), resulting in a relevant glucose contribution to increased liver lipogenesis and thus local and ectopic lipid deposition.

## 5. Conclusions 

In conclusion, our study clearly supports that, in adult age, neonatally damaged MSG male rats are overall insulin-resistant and with increased OS (at both peripheral and liver levels). These features are accompanied by a dramatic dysfunction in hepatic metabolism: enhanced glucose cycling (GCK/G6Pase activities) and increased pentose phosphate shunt, together with adaptive changes in FK activity. Indeed, MSG rats displayed several features characteristic of the human Cushing's and metabolic syndrome phenotypes, including a decreased liver weight, high tissue inflammation, and local metabolism highly displaced to an increase in lipid production.

It remains to be determined whether the MSG liver dysfunction can be ameliorated by correcting endogenous glucocorticoid [14, 18], insulin [18, 60], and leptin [14, 18] overproduction. Finally, the risk factor of MSG liver imbalance in the acyl-ceramidase/ceramide [61] cycle, a major cooperative mechanism responsible for development of hepatic steatosis, requires further investigation.

## Figures and Tables

**Table 1:** Rat specific primers used for real-time PCR analyses.

		GBAN	bp
*Actb*	F, 5′-AGAGGGAAATCGTGCGTGAC-3′	NM_031144	138
	R, 5′-CGATAGTGATGACCTGACCGT-3′		
*Chrebp*	F, 5′-CAGATGCGGGACATGTTTGA-3′	NM_133552.1	205
	R, 5′-AATAAAGGTCGGATGAGGATGCT-3′		
*Fas*	F, 5′-GTCTGCAGCTACCCACCCGTG-3′	NM_017332.1	214
	R, 5′-CTTCTCCAGGGTGGGGACCAG-3′		
*Fk*	F, 5′-ACGGATCGCAGGTGCCTAT-3′	NM_031855.3	68
	R, 5′-AGCACAGTGCAGGAGTTGGA-3′		
*Gck*	F, 5′-GTGTACAAGCTGCACCCGA-3′	NM_012565.1	156
	R, 5′-CAGCATGCAAGCCTTCTTG-3′		
*Gpat*	F, 5′-GACGAAGCCTTCCGAAGGA-3′	AF_021348	68
	R, 5′-GACTTGCTGGCGGTGAAGAG-3′		
*G6pase*	F, 5′-GATCGCTGACCTCAGGAACGC-3′	NM_013098.2	198
	R, 5′-AGAGGCACGGAGCTGTTGCTG-3′		
*G6pdh*	F, 5′-TTCCGGGATGGCCTTCTAC-3′	NM_017006.2	81
	R, 5′-TTTGCGGATGTCATCCACTGT-3′		
*Il1b*	F, 5′-ACAAGGAGAGACAAGCAACGAC-3′	NM_031512.2	140
	R, 5′-TCTTCTTTGGGTATTGTTTGGG-3′		
*Pai1*	F, 5′-CCACGGTGAAGCAGGTGGACT-3′	NM_012620.1	195
	R, 5′-TGCTGGCCTCTAAGAAGGGG-3′		
*Pepck*	F, 5′-TGCCCCAGGAAGTGAGGAAG-3′	NM_198780.3	177
	R, 5′-GGTCAGTGAGAGCCAGCCAAC-3′		
*Pfk2*	F, 5′-CGATCTATCTACCTATGCCGCCAT-3′	NM_012621.4	256
	R, 5′-ACACCCGCATCAATCTCATTCA-3′		
*Srebp1c*	F, 5′-TTTCTTCGTGGATGGGGACT-3′	XM_213329.5	208
	R, 5′-CTGTAGATATCCAAGAGCATC-3′		
*Tnfa*	F, 5′- GGCATGGATCTCAAAGACAACC-3′	NM_012675.3	130
	R, 5′- CAAATCGGCTGACGGTGTG-3′		

F: forward primer; R: reverse primer; GBAN: GenBank accession number; amplicon length, in bp.

**Table 2:** Body weight (BW), daily food intake, wet tissue (visceral adipose tissue, VAT, and liver), and mass and peripheral levels of several biomarkers in control (C) and MSG rats.

	C	MSG
Body weight (g)	404.6 ± 9.1	349.3 ± 7.6^*∗*^
Food intake (g/day)	20.81 ± 1.95	15.61 ± 0.87^*∗*^
VAT mass (g)	4.47 ± 0.88	14.18 ± 1.77^*∗*^
Liver weight (g/100 g BW)	3.19 ± 0.07	2.93 ± 0.06^*∗*^
Glycemia (g/L)	1.06 ± 0.02	1.17 ± 0.03
Insulin (ng/mL)	0.81 ± 0.05	1.37 ± 0.19^*∗*^
Leptin (ng/mL)	1.39 ± 0.37	16.49 ± 2.38^*∗*^
Triglycerides (g/L)	1.06 ± 0.07	1.85 ± 0.18^*∗*^
Uric acid (mg/dL)	1.19 ± 0.09	1.82 ± 0.22^*∗*^
Corticosterone (*μ*g/dL)	6.19 ± 0.98	13.75 ± 1.12^*∗*^
GOT (U/L)	75.5 ± 3.2	96.9 ± 5.8^*∗*^
GPT (U/L)	9.51 ± 0.69	15.49 ± 1.19^*∗*^

Values are means ± SEM. ^*∗*^ *P* < 0.05 versus C values (*n* = 12 rats per group).

**Table 3:** Peripheral and liver (*n* = 12 and 6 specimens per group, resp.) oxidative stress markers in control (C) and MSG rats.

	C	MSG
Peripheral TBARS (pmol/mg of protein per mL of plasma)	40.3 ± 3.6	85.1 ± 15.2^*∗*^
Liver protein carbonyl groups (nmol/mg of tissue protein)	4.43 ± 0.15	7.81 ± 1.32^*∗*^
Liver GSH (nmol/g tissue)	2.71 ± 0.03	2.05 ± 0.04^*∗*^

Values are means ± SEM. ^*∗*^ *P* < 0.05 versus C values.
