# Corrigendum to “Chronic Glucocorticoid-Rich Milieu and Liver Dysfunction”

**DOI:** 10.1155/2018/1239718

**Published:** 2018-10-23

**Authors:** Hernán Gonzalo Villagarcía, Vanesa Sabugo, María Cecilia Castro, Guillermo Schinella, Daniel Castrogiovanni, Eduardo Spinedi, María Laura Massa, Flavio Francini

**Affiliations:** ^1^Centro de Endocrinología Experimental y Aplicada (CENEXA), UNLP-CONICET-FCM, 1900 La Plata, Argentina; ^2^Cátedra de Farmacología Básica, Facultad de Ciencias Médicas, UNLP and CICPBA, 1900 La Plata, Argentina; ^3^Instituto Multidisciplinario de Biología Celular (IMBICE), CONICET-CICPBA-UNLP, 1900 La Plata, Argentina

In the article titled “Chronic Glucocorticoid-Rich Milieu and Liver Dysfunction” [1], there was an error in Table 3 where the units for GSH should have been those that were mentioned in the “Materials and Methods” under the “Liver Protein Carbonyl Groups and Reduced Glutathione (GSH).” Therefore, the unit should be corrected from nmol/g tissue to *μ*mol/g tissue. The corrected table is as follows.

## Figures and Tables

**Table 3:** Peripheral and liver (*n* = 12 and 6 specimens per group, resp.) oxidative stress markers in control (C) and MSG rats.

	C	MSG
Peripheral TBARS (pmol/mg of protein per mL of plasma)	40.3 ± 3.6	85.1 ± 15.2^∗^
Liver protein carbonyl groups (nmol/mg of tissue protein)	4.43 ± 0.15	7.81 ± 1.32^∗^
Liver GSH (*μ*mol/g tissue)	2.71 ± 0.03	2.05 ± 0.04^∗^

Values are means ± SEM. ^∗^ *P* < 0.05 versus C values.
